# Stentolith in Bile Duct: A Neglected Entity—Case Report with Review of Literature

**DOI:** 10.1055/s-0042-1743521

**Published:** 2022-03-03

**Authors:** Amit Gupta, Deepak Rajput, Jaine John Chennat, Tanuj Singla, Shaik Sameer Ahmed

**Affiliations:** 1 Department of Surgery, All India Institute of Medical Sciences, Rishikesh, Uttarakhand, India

**Keywords:** stentolith, cholelithiasis, stenting, cholangiography

## Abstract

Stentolith is a forgotten stent that acts as a nidus for stone formation leading to a stone-stent complex. Once the planned procedure is completed, these stents should be removed within 4 to 6 weeks, but if they are required for a longer period, then they should be replaced every 3 to 6 months. Devastating complications may ensue —such as cholangitis, biliary stricture, or secondary biliary cirrhosis. Management primarily comprises surgical intervention with common bile duct exploration or endoscopic clearance. The majority of patients eventually develop symptoms that lead to their diagnosis and subsequent management. This article, however, details the case of a silent stentolith and how it may have led to disastrous complications if surgical intervention was not done promptly.

Choledocholithiasis occurs in 10 to 15% of patients with cholelithiasis. 1 Endoscopic retrograde cholangiopancreatography with common bile duct (CBD) stone clearance and/or CBD stenting followed by laparoscopic cholecystectomy or laparoscopic CBD exploration with cholecystectomy as a single-stage surgery is the current mainstay of treatment of cholelithiasis with choledocholithiasis. 2

Stentolith is essentially a complication of a forgotten stent in the CBD which acts as a nidus for stone formation. 3 The sequelae of a retained or forgotten stent in the CBD include choledocholithiasis, biofilm formation, and bacterial adhesion to the stent leading to ascending infection or cholangitis, occlusion, or migration of the stent. 1 4 5

In most instances, patients with forgotten stents present with symptoms such as abdominal pain, jaundice, or fever which lead to their subsequent diagnosis and management. However, asymptomatic patients who are unaware of their condition pose a unique diagnostic dilemma as the diagnosis is made only on routine examination or when the condition becomes grave enough for the patient to become symptomatic—such as the development of complications such as cholangitis.

## Case Report

**Fig. 1:**
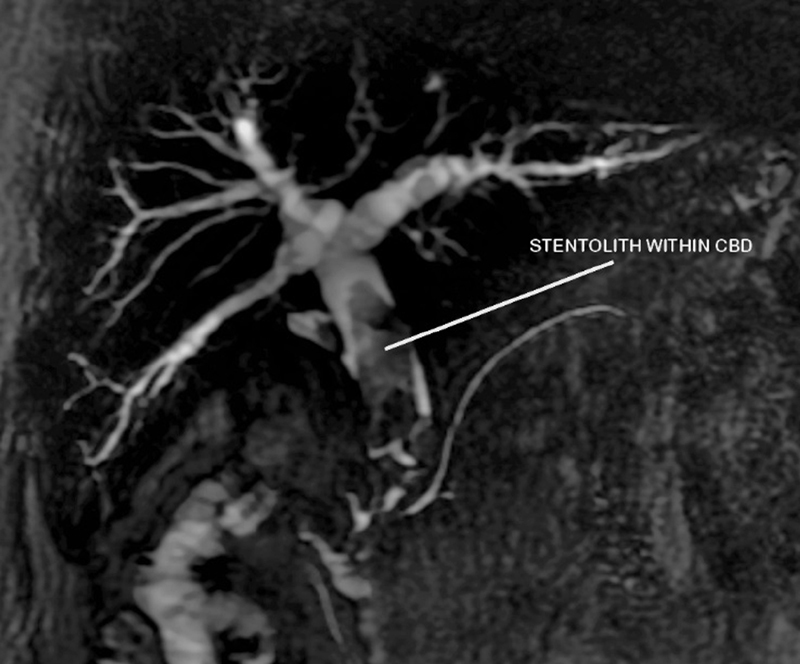
MRCP image showing Stentolith in common bile duct.

**Fig. 2:**
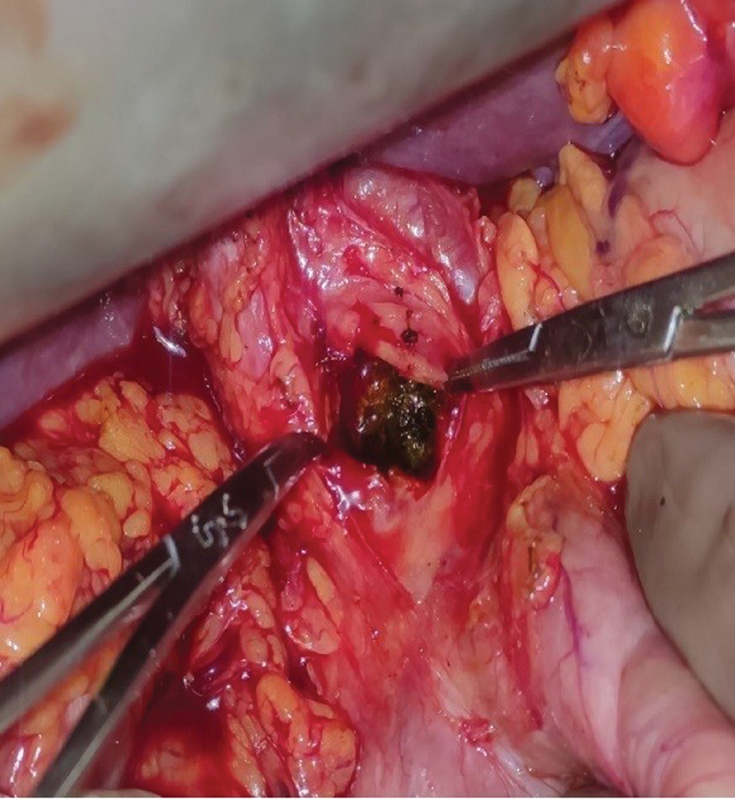
Intraoperative figure depicting the Stentolith.

**Fig. 3:**
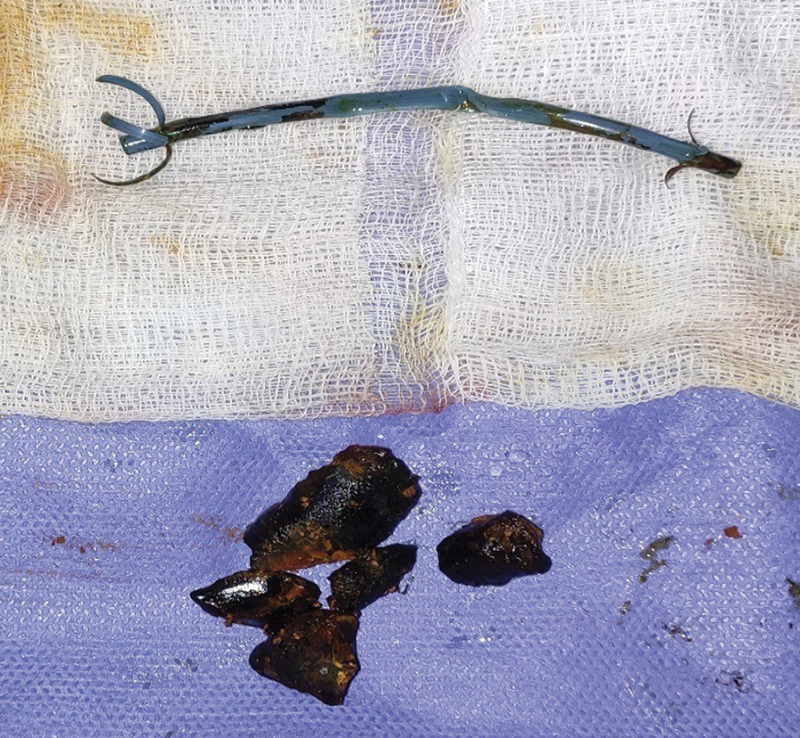
Stone-stent complex after removal.

A 51-year-old man presented with a history of cholelithiasis and choledocholithiasis in 2015 for which he underwent endoscopic retrograde cholangiopancreatography (ERCP) with CBD stenting followed by laparoscopic cholecystectomy in the same hospital stay. History was significant for type 2 diabetes mellitus for which he was on irregular medication. He was then lost to follow up. Five years later, he presented to our institute for a routine checkup—he was asymptomatic. The laboratory investigations were as follows: alkaline phosphatase—414.4 U/L, gamma glutamyl transferase—450.2 U/L, aspartate transaminase—44.3 U/L, alanine transaminase—63.7 U/L, total bilirubin—1.61 mg/dL, direct bilirubin—0.94 mg/dL, serum protein—7.17 g/dL, serum albumin—4.16 g/dL, and prothrombin time/international normalized ratio—19.2/1.44. The rest of the investigations were within normal limits. An ultrasound of the abdomen was done which reported a filling defect in CBD. Magnetic resonance cholangiopancreatography was done which was suggestive of CBD stone possibly stentolith of size 4 × 1.2 cm with dilated CBD (8 mm). In axial cuts, bile was noted to be passing through the stentolith ( Fig. 1 ). This radiological finding possibly accounted for the lack of obstructive symptoms such as jaundice or cholangitis. Thus, if undetected, the stone could have progressed in size, eventually leading to obstruction with its associated complications. Preoperatively, patient was subjected to ERCP, but could not be retrieved as it was impacted in the bile duct. He, therefore, underwent open CBD exploration because of the large size of the stone and expected dense adhesions due to previous surgery. Intraoperatively, a 5 × 1.2-cm brown pigmented stone encasing the CBD stent was noted ( Fig. 2 ). The “stentolith” and surrounding small fragmented CBD stone clearance were done ( Fig. 3 ). On further inspection of the CBD stent, luminal patency was not present. No cystic duct remnant or pancreatic duct calculi was felt on palpation. The postoperative period was uneventful; the patient was stable; bile cultures were sterile. A check T-tube cholangiography done on postoperative day 14 was normal and T-tube was removed. On routine follow-up, the patient was doing well with no complications.

## Discussion

Choledocholithiasis is seen in ∼10 to 15% of patients with cholelithiasis. 6 The majority of choledocholithiasis cases are secondary; primary choledocholithiasis is a relatively rare entity but seen more frequently in persons of Asian descent. Primary CBD stones, formed initially in the bile ducts, are primarily due to biliary stasis and bacterial or parasitic infection. Secondary stones, on the other hand, originate in the gallbladder and pass into the bile duct. 7 In 1974, the introduction of endoscopic diagnostic and therapeutic modalities, that is, ERCP with sphincterotomy and CBD stenting ushered a new era in the management of the biliary stone disease. 8 The current mainstay of treatment for cholelithiasis with choledocholithiasis includes ERCP with CBD stone clearance ± endoscopic sphincterotomy ± CBD stenting followed by laparoscopic cholecystectomy if feasible. For larger stones, open or laparoscopic CBD exploration is done. 2 The CBD stents used are of two broad categories—plastic and metallic stents. Metallic stents are usually made of stainless steel or nickel–titanium alloy. Plastic stents are generally used as a temporary measure—for post-ERCP CBD clearance, palliative stenting in metastatic disease with an expected life span < 3 months, or temporary biliary drainage before surgery. Plastic stents, although economical, are prone to getting occluded—primarily due to complexes formed of microbial colonies and bacterial byproducts coupled with calcium bilirubinate and calcium palmitate crystals which eventually promote bacterial adherence and biofilm formation. The release of bacterial β-glucuronidase also plays a role by causing precipitation of calcium bilirubinate which is aggregated into stones by glycoproteins. The retained stent also acts as a foreign body, thereby promoting colonization of the bacteria over it. 9 10 Thus, plastic stents for choledocholithiasis should ideally be removed within 3 to 6 months of insertion of the stent to avoid the myriad of complications such as cholangitis, stentolith formation, or bile duct stricture. 11 However, many patients, either due to ignorance or lack of education about the endoprosthesis, fail to follow up in time for the stent removal. 12 Thus, instead of undergoing CBD stent removal, a seemingly benign procedure, the patient ends up undergoing a complicated surgery with the potential for more morbidity. The sequelae of the forgotten biliary endoprosthesis are highly varied—it may remain silent for years or may have devastating complications. In symptomatic patients, common clinical presentations include jaundice, abdominal pain, vomiting, and fever. The most common complications according to some case series include choledocholithiasis and stentolith formation. Others include severe cholangitis, stent migration, stent occlusion or fracture, biliary stricture, or secondary biliary cirrhosis. 2 10 The majority of the patients will require surgical intervention for the clearance of stentoliths—whether laparoscopic or open CBD exploration depending on the size and adherence of the stone and the surgeons' preference. Bilioenteric bypass with side-to-side Roux-en-Y choledochojejunostomy, CBD exploration with choledochoduodenostomy, and endoscopic clearance of stentoliths have also been done in some cases. 13 14 15 16

## Conclusions

- A forgotten biliary stent can have devastating complications on the patients' life.
- It seems inconceivable that a person can forget that they have a stent that must be removed; however, many factors may play a role in this entity.
- Lack of awareness on the side of the patient due to inadequate counseling, the presumption that the stent is permanent, lack of proper documentation, noncompliance with follow-up, lower socioeconomic status, lack of follow-up due to geographical difficulties, and rapid resolution of symptoms paired with lack of operative scar are all factors that result in delayed presentation.
- “Biliary stent registries,” as mentioned by Bansal et al, by a dedicated team should be instituted to decrease noncompliance of timely follow-up.
- Thus, there is an urgent need for the education and robust counseling of patients undergoing ERCP with CBD stenting which would have significant implications in the reduction of morbidity of this preventable disease entity.

## References

[1] Barai V Hedawoo J Changole S Forgotten CBD stent (102 months) with stone-stent complex: a case report Int J Surg Case Rep 2017 30 162 164 28012336 10.1016/j.ijscr.2016.11.048 PMC5198634

[2] Odabasi M Arslan C Akbulut S Long-term effects of forgotten biliary stents: a case series and literature review Int J Clin Exp Med 2014 7 08 2045 2052 25232385 PMC4161545

[3] Bansal V K Misra M C Bhowate P Kumar S Laparoscopic management of common bile duct “Stentolith” Trop Gastroenterol 2009 30 02 95 96 19760992

[4] Leung J W Sung J Y Costerton J W Bacteriological and electron microscopy examination of brown pigment stones J Clin Microbiol 1989 27 05 915 921 2745700 10.1128/jcm.27.5.915-921.1989 PMC267454

[5] Srinivasan I Kahaleh M Biliary stents in the millennium Adv Ther 2011 28 11 960 972 21984349 10.1007/s12325-011-0067-4

[6] McNicoll C F Pastorino A Farooq U St Hill C R Choledocholithiasis Treasure Island (FL) StatPearls Publishing 2020 28722990

[7] Ko C W Lee S P Epidemiology and natural history of common bile duct stones and prediction of disease Gastrointest Endosc 2002 56(6, Suppl):S165 S169 12447261 10.1067/mge.2002.129005

[8] Yoo K S Lehman G A Endoscopic management of biliary ductal stones Gastroenterol Clin North Am 2010 39 02 209 227 20478483 10.1016/j.gtc.2010.02.008

[9] Donelli G Guaglianone E Di Rosa R Fiocca F Basoli A Plastic biliary stent occlusion: factors involved and possible preventive approaches Clin Med Res 2007 5 01 53 60 17456835 10.3121/cmr.2007.683 PMC1855334

[10] Kumar S Chandra A Kulkarni R Maurya A P Gupta V Forgotten biliary stents: ignorance is not bliss Surg Endosc 2018 32 01 191 195 28643071 10.1007/s00464-017-5657-z

[11] European Society of Gastrointestinal Endoscopy Dumonceau J-M Tringali A Blero D Biliary stenting: indications, choice of stents and results: European Society of Gastrointestinal Endoscopy (ESGE) clinical guideline Endoscopy 2012 44 03 277 298 22297801 10.1055/s-0031-1291633

[12] Duman A E Yılmaz H Hülagü S Biliary stents are forgotten more frequently in elderly patients Turk J Med Sci 2021 51 06 3067 3072 34579509 10.3906/sag-2104-108 PMC10734834

[13] Kumar S Chandra A Giant stentolith: a rare complication of long-dwelling biliary endoprosthesis Arab J Gastroenterol 2020 21 02 132 134 32423857 10.1016/j.ajg.2020.04.008

[14] Bajbouj M Treiber M Ludwig L Frimberger E Schmid R M Neu B Forgotten biliary endoprosthesis. “Follow up” after 10 years Endoscopy 2008 40(02, Suppl 2):E221 18819066 10.1055/s-2008-1077431

[15] Gupta V Chandra A Noushif M Singh S K Giant stentolith: complication of a forgotten biliary stent Endoscopy 2013 45(2, suppl 2 UCTN)E126 23716092 10.1055/s-0032-1326367

[16] Patel T J Rajput S Patel K S Haribhakti S P A forgotten biliary stent for 17 years: presented with perforated gallbladder and stentolith J Dig Endosc 2014 05 01 22 23

